# Metformin Use Is Associated with Decreased Mortality in COVID-19 Patients with Diabetes: Evidence from Retrospective Studies and Biological Mechanism

**DOI:** 10.3390/jcm10163507

**Published:** 2021-08-09

**Authors:** Tahmina Nasrin Poly, Md. Mohaimenul Islam, Yu-Chuan (Jack) Li, Ming-Chin Lin, Min-Huei Hsu, Yao-Chin Wang

**Affiliations:** 1 Graduate Institute of Biomedical Informatics, College of Medical Science and Technology, Taipei Medical University, Taipei 110301, Taiwan; d610108004@tmu.edu.tw (T.N.P.); d610106004@tmu.edu.tw (M.M.I.); jack@tmu.edu.tw (Y.-C.L.); arbiter@tmu.edu.tw (M.-C.L.); 2 International Center for Health Information Technology (ICHIT), Taipei Medical University, Taipei 110301, Taiwan; 3 Research Center of Big Data and Meta-Analysis, Wan Fang Hospital, Taipei Medical University, Taipei 110301, Taiwan; 4 Department of Dermatology, Wan Fang Hospital, Taipei 116081, Taiwan; 5 TMU Research Center of Cancer Translational Medicine, Taipei Medical University, Taipei 110301, Taiwan; 6 Department of Neurosurgery, Shuang Ho Hospital, Taipei Medical University, Taipei 110301, Taiwan; 7 Professional Master Program in Artificial Intelligence in Medicine, Taipei Medical University, Taipei 110301, Taiwan; 8 Graduate Institute of Data Science, Taipei Medical University, Taipei 110301, Taiwan; 701056@tmu.edu.tw; 9 Department of Emergency, Min-Sheng General Hospital, Taoyuan 33044, Taiwan; 10 Graduate Institute of Injury Prevention and Control, College of Public Health, Taipei Medical University, Taipei 11031, Taiwan

**Keywords:** metformin, diabetes, insulin, mortality, severity, COVID-19, meta-analysis

## Abstract

Background and Aims: The coronavirus disease 2019 (COVID-19) increases hyperinflammatory state, leading to acute lung damage, hyperglycemia, vascular endothelial damage, and a higher mortality rate. Metformin is a first-line treatment for type 2 diabetes and is known to have anti-inflammatory and immunosuppressive effects. Previous studies have shown that metformin use is associated with decreased risk of mortality among patients with COVID-19; however, the results are still inconclusive. This study investigated the association between metformin and the risk of mortality among diabetes patients with COVID-19. Methods: Data were collected from online databases such as PubMed, EMBASE, Scopus, and Web of Science, and reference from the most relevant articles. The search and collection of relevant articles was carried out between 1 February 2020, and 20 June 2021. Two independent reviewers extracted information from selected studies. The random-effects model was used to estimate risk ratios (RRs), with a 95% confidence interval. Results: A total of 16 studies met all inclusion criteria. Diabetes patients given metformin had a significantly reduced risk of mortality (RR, 0.65; 95% CI: 0.54–0.80, *p* < 0.001, heterogeneity I^2^ = 75.88, Q = 62.20, and τ^2^ = 0.06, *p* < 0.001) compared with those who were not given metformin. Subgroup analyses showed that the beneficial effect of metformin was higher in the patients from North America (RR, 0.43; 95% CI: 0.26–0.72, *p* = 0.001, heterogeneity I^2^ = 85.57, Q = 34.65, τ^2^ = 0.31) than in patients from Europe (RR, 0.67; 95% CI: 0.47–0.94, *p* = 0.02, heterogeneity I^2^ = 82.69, Q = 23.11, τ^2^ = 0.10) and Asia (RR, 0.90; 95% CI: 0.43–1.86, *p* = 0.78, heterogeneity I^2^ = 64.12, Q = 11.15, τ^2^ = 0.40). Conclusions: This meta-analysis shows evidence that supports the theory that the use of metformin is associated with a decreased risk of mortality among diabetes patients with COVID-19. Randomized control trials with a higher number of participants are warranted to assess the effectiveness of metformin for reducing the mortality of COVID-19 patients.

## 1. Introduction

### 1.1. Rationale

On 31 December 2019, the first outbreak of coronavirus diseases-2019 (COVID-19) started in Wuhan, China, and has since affected more than 220 countries worldwide [1]. There are no specific drugs against SARS-CoV-2 infection; however, several existing drugs have been used to manage the disease’s severity [2]. As of 7 March 2021, the total number of confirmed cases has exceeded 116 million, and the total number of deaths is more than 2.5 million (https://www.worldometers.info/coronavirus/, accessed on 5 August 2021). Previous studies have reported that patients with multiple conditions, including diabetes, hypertension, obesity, and cardiac disease, are often at increased risk of acute respiratory distress syndrome (ARDS) and mortality [3,4,5,6]. Recently, observational studies have demonstrated that metformin use, both before and after diagnosis of COVID-19, is associated with a substantially decreased risk of mortality among patients with COVID-19 [7,8].

Several biological mechanisms can explain the potential biological effect of metformin on COVID-19 mortality. First, the mortality rate for COVID-19 is substantially higher in patients with an uncontrolled glucose level. Metformin is a widely used hypoglycemic drug and helps to improve the outcome of COVID-19 patients with diabetes by controlling glucose levels. Second, metformin activates adenosine monophosphate-activated protein kinase (AMPK), which eventually increases mitochondrial metabolism and autophagy and lessens the level of the inflammatory factors [9]. Third, SARS-CoV-2 induces the secretion of interferon-gamma and increases muscular insulin resistance and circulating insulin levels, which eventually increases the response of cluster of differentiation 8 cytotoxic T-cell (CD8 + T-cell) [10]. However, metformin has a protective role in the mitochondrial electron transport chain [11] and impairs memory T-cell responses through glycolysis promotion [12]. Given the epidemiological and biological plausibility of the benefits, there is an unmet need of meta-analysis to evaluate the magnitude of the association between them.

### 1.2. Goal of Investigation

Currently, significant data are available in retrospective studies concerning the beneficial effects of metformin on COVID-19 that could be used to facilitate personalized decision making in patients with COVID-19. Therefore, systematically reviewed evidence from existing retrospective studies to clarify the association between metformin use and the risk of mortality among patients with COVID-19.

### 1.3. Hypothesis

The research questions were:

- Magnitude of the risk of mortality among COVID-19 patients with metformin and those without metformin;
- Difference of magnitude of the risk of mortality among COVID-19 patients in the various continents.

## 2. Methods

This study was deemed exempt from review by the Taipei Medical University Review Board. No patient informed consent was required. The PRISMA (Preferred Reporting Items for Systematic Reviews and Meta-Analyses) guidelines, which are based on the Cochrane Handbook for Systematic Reviews of Interventions, were used to conduct this study [13,14,15].

### 2.1. Search Strategy

A comprehensive and systematic search was conducted in online databases such as PubMed, Scopus, Embase, and Web of Science between 1 February 2020 and 20 June 2021, with no restriction of language. The keywords used to search the most relevant articles were: “Metformin” and “coronavirus related mortality” OR “COVID-19 mortality” OR “SARS-CoV-2 virus mortality”.

### 2.2. Eligibility Criteria

Study were included if they: (a) were restricted to epidemiological studies and evaluated the risk of mortality among COVID-19 patients with or with metformin, (b) included at least 15 participants to calculate the effect size, (c) provided clear inclusion and exclusion criteria of COVID-19 patients and metformin exposure. Studies were excluded if they had been published in the form of an editorial or review. Furthermore, we selected the most recent studies (hypothesized metformin and COVID-19 mortality) if they used similar databases.

### 2.3. Data Extraction

Two reviewers (M.M.I. and T.N.P.) independently screened the retrieved articles on the basis of pre-specified inclusion and exclusion criteria. Any disagreement between them was ultimately resolved through discussion with the main investigator. They first screened all the titles and abstracts, and the most relevant articles were kept for full-text revision. If the same author had published multiple papers using the same database, then the most recent study was considered for inclusion.

### 2.4. Statistical Analysis

The same two authors collected the effect size in term of the hazard ratio (HR) or odds ratio (OR) for each study, with a 95% CI. The random-effect model was used to calculate the risk ratio (RR) for the outcome of interest (COVID-19 mortality), with 95% CI. The I^2^ and Q statistics were also calculated to measure heterogeneity. The I^2^ value was also classified into four groups (0~25: very low, 25~50: low, 50~75: moderate, and >75: high). Forest plots were drawn to show the effect size for all associations. A funnel plot was constructed to present publication bias. However, all analyses were conducted using statistical software (Comprehensive Meta-Analysis, version 2.0, Biostat Inc. 14 North Dean Street, Englewood, NJ, USA).

## 3. Results

### 3.1. Literature Search

The online databases search yielded 228 articles. After reviewing all the titles and abstracts, 205 were excluded. A total of 23 articles went for full-text review, and 16 articles finally met all inclusion criteria [7,8,16,17,18,19,20,21,22,23,24,25,26,27,28,29] (Supplementary Figure S1).

### 3.2. Study Characteristics

Table 1 shows the baseline characteristics of the included studies. All of the studies used a retrospective study design. The percentage of male patient was between 35.1 and 97.3. Six studies were conducted in North America, five studies were from Europe, and five studies were from Asia. All patients had type-2 diabetes, and the number of metformin users were between 9 and 1,800,005. All studies reported in-hospital mortality, except for one.

### 3.3. Primary Analysis

#### 3.3.1. Metformin Use and COVID-19 Mortality

**Figure 1:**
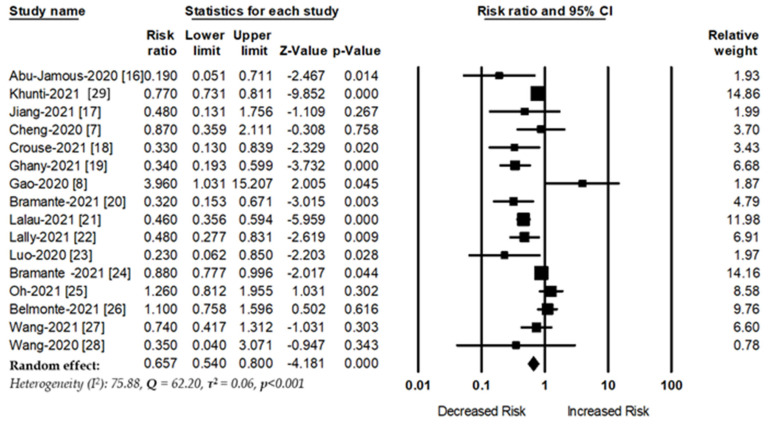
Association between metformin use and COVID-19 mortality.

A total of 16 studies assessed the association between metformin use and the risk of mortality among patients with COVID-19. Metformin use was associated with a 35% decreased in risk of mortality among patients with COVID-19 (RR, 0.65, 95%CI: 0.54–0.80, *p* < 0.001) (Figure 1). There was a significant heterogeneity between the studies (I^2^ = 75.88, Q = 62.20, and τ^2^ = 0.06, *p* < 0.001).

#### 3.3.2. Subgroup Analysis

Subgroup analyses were also conducted to examine the magnitude of their association from different perspectives (Table 2). Eight studies used a nation-wide database, and eight studies used hospital-based data to evaluate the risk of COVID-19 mortality among patients with metformin. The overall risk of COVID-19 mortality with metformin were RR, 0.74 (95%CI: 0.60–0.89) and RR, 0.45 (95%CI: 0.27–0.74), respectively.

There were no associations between metformin and COVID-19 mortality in the Asian population (RR, 0.90, 95%CI: 0.43–1.86). However, the beneficial effect of metformin was observed in the patients from Europe and North America (RR, 0.67, 95%CI: 0.47–0.94 vs. RR, 0.43, 95%CI: 0.26–0.72).

#### 3.3.3. Sensitivity Analysis

To confirm the robustness of our findings, we categorized studies into three groups based on the number of metformin users with COVID-19 (<1000, 1000–10,000, and >10,000). Eleven studies included less than 1000 metformin users, and the risk of COVID-19 related mortality was significantly lower among metformin users (RR, 0.53 (95%CI: 0.32–0.81, *p* = 0.004)) (Supplementary Figure S2). There was a moderate significant heterogeneity among the studies (I^2^ = 69.31, Q = 32.58, and τ^2^ = 0.35, *p* < 0.001). Three studies included metformin users between 1000 and 10,000 and there was a insignificant reduction in mortality among metformin users (RR, 0.78 (95%CI: 0.47–1.29, *p* = 0.34)) (Supplementary Figure S3). There was a higher significant heterogeneity among the studies (I^2^ = 91.80, Q = 24.40, and τ^2^ = 0.17, *p* <0.001). Two studies included more than 10,000 metformin users with COVID-19, and there was a significant reduction in mortality among metformin users (RR, 0.77 (95%CI: 0.73–0.81, *p* < 0.001)) (Supplementary Figure S4). There was no heterogeneity between the studies (I^2^ = 0.00, Q = 0.01, and τ^2^ = 0, *p* = 0.89).

### 3.4. Secondary Analysis:

#### Metformin Use and Acute Respiratory Distress Syndrome Risk

**Figure 2:**
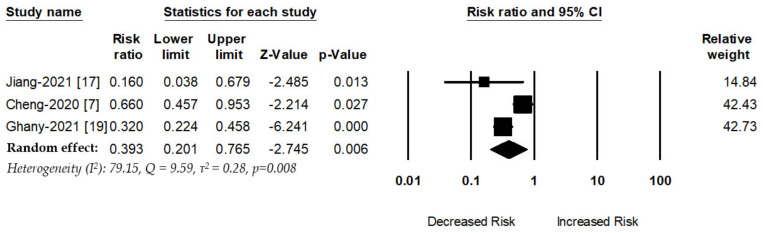
Association between metformin use and ARDS risk.

Three studies evaluated the association between metformin use and Acute Respiratory Distress Syndrome (ARDS) risk. The overall pooled effect shows that metformin use was associated with a decreased risk of ARDS among COVID-19 patients with diabetes (RR, 0.39; 95%CI: 0.20–0.76, *p* = 0.006, I^2^ = 79.15, Q = 9.59, τ^2^ = 0.28) (Figure 2).

### 3.5. Publication Bias

**Figure 3:**
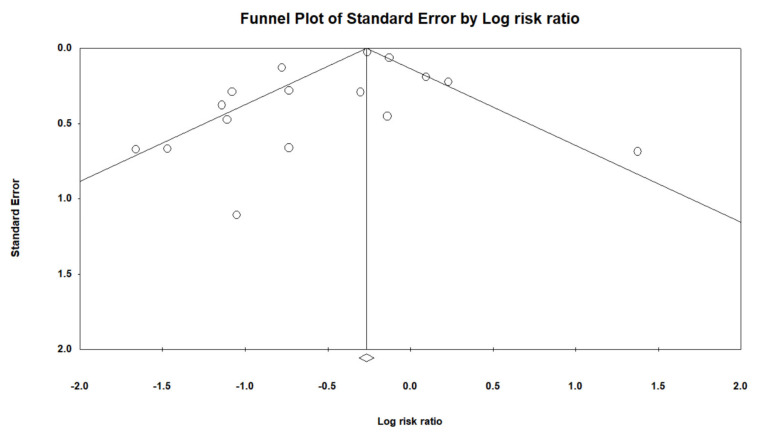
Funnel plot.

In Figure 3, the funnel plot shows no publication bias between the studies. Egger’s regression test was utilized to assess the funnel asymmetry, which indicated no publication bias (*p* = 0.20).

## 4. Discussion

Our meta-analysis was designed to clarify the association between metformin use and risk of mortality in patients with COVID-19. The findings of our study show that metformin was associated with a decreased risk of mortality among COVID-19 patients, both before and after use. The beneficial effects of metformin was lower in the Asian population than in the non-Asian counterparts (North American and European). Genetic susceptibility and variation of β-cell function may influence their response to metformin treatment [30,31].

Our study findings are pertinent with four previous meta-analysis (Table 3) [32,33,34,35]. Lukito et al. [35] aimed to show the positive effect of metformin use on mortality in hospitalized COVID-19 patients, and nine studies met inclusion criteria. Metformin use was associated with a 36% reduced risk of mortality among hospitalized COVID-19 patients. However, they did not provide any subgroup analysis and sensitivity analysis. Scheen et al. [34] conducted a meta-analysis using only four studies, showing that metformin consumption was associated with a reduced risk of mortality among patients with COVID-19 (OR, 0.75, 95% CI: 0.67–0.85).

**Figure 4:**
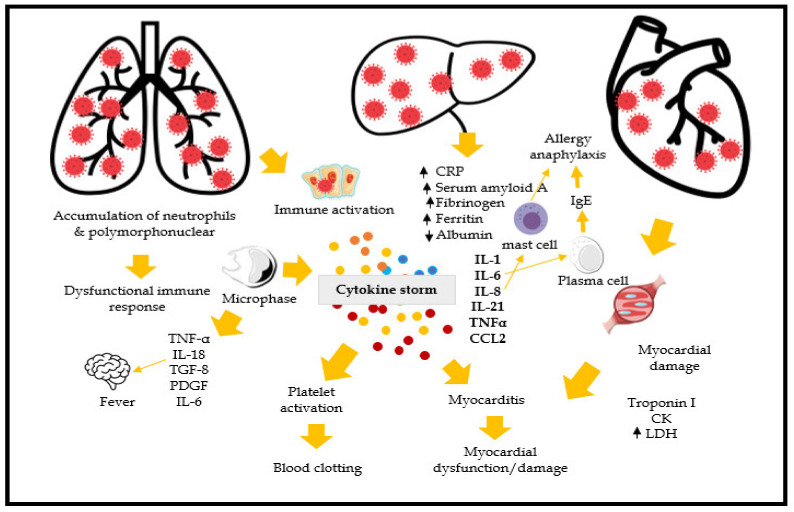
Biological mechanism of COVID-19. (Note: CRP: C-reactive protein; CCL2: chemokine ligand 2; TNF- α: tumor necrosis factor-α; IL-1, 6, 8, 18, 21: Interleukin-1, 6, 8, 18, 21; TGF-8: Transforming growth factor 8; PDGF: Platelet-derived growth factor; LDH: Lactate dehydrogenase, IgE: Immunoglobulin E).

Several biological possibilities can explain the beneficial mechanism of metformin in COVID-19 patients (Table 4). Previous studies have shown that tumor necrosis factor-α (TNFα) has a significant role in COVID-19 pathology; it activates macrophage, increases cytokine release, and worsens the patient’s condition (Figure 4). However, metformin helps decrease cytokine release [Interleukin 6 (IL-6), TNFα], decreases thrombosis, reduces glycaemia, and increases the neutrophil to lymphocyte ration through angiotensin-converting enzyme 2 (ACE2) receptor modulation. There was a reduced level of inflammatory mediators, IL-6 and TNFα, in both diabetes and non-diabetes patients while using metformin. Moreover, metformin shows the significant positive effect on reducing the neutrophil counts, and decreasing neutrophil extracellular traps [36,37]. Metformin helps to increase ACE-2 expression via adenosine monophosphate-activated protein kinase (AMPK) activation, which leads to reduced cytokine response. It is reported that the direct entry of SAR-CoV-2 increase endoplasmic reticulum stress; however, metformin suppress the ER stress through activation of the 5-AMPK-phosphatidylinositol 3 kinase (PI3K)-c-Jun NH2 pathway [38].

In addition, metformin help to reduce the release of inflammatory markers by affecting the MTOR and NF-kappa B pathways [39]. Previous studies have demonstrated that SARS-CoV-2 activates several cellular responses, including cellular stress responses such as unfolded protein response (UPR) and autophagy, through the inhibition of mTOR [40]. The biological mechanism of UPR and autophagy are involved in cellular and tissue homeostasis, apoptosis, and innate immunity modulation. However, metformin has great potential to inhibit protein synthesis, inhibit UPR, and activate the immune system [41]. It also appears that the physical condition of patients with SARS-CoV-2 is more likely to deteriorate due to multiple comorbidities, including cardiovascular diseases. Invasion of SARS-CoV-2 is associated with advanced vascular endothelial glycocalyx damage, especially in elderly patients [42]. However, deterioration of vascular endothelial glycocalyx can be a potent mechanism for the development of life-threatening complications, including acute kidney injury, among COVID-19 patients [43]. Multiples studies have shown that metformin induces endothelial glycocalyx restoration and protects the cardiovascular system [44,45].

Other molecular pathways common to diabetes and SARS-CoV-2 infection can be used to explain the potential benefit of metformin. Viral-induced interferon-gamma secretion has been demonstrated to increase muscular insulin resistance and circulating insulin levels, which, in turn, increases the cluster of differentiation 8 cytotoxic T-cell (CD8 + T-cell) responses.

Our study has several strengths. First, it is an updated meta-analysis of 17 studies that evaluated the beneficial effect of metformin on COVID-19 mortality. Second, we have shown a broad subgroup analysis of the association between them. Third, we have shown several possible mechanisms of how metformin plays a protective role in COVID-19 mortality.

Our study has some limitations that need to be addressed. First, our study shows metformin was associated with a decreased risk of mortality but the duration of metformin use and reduced risk of mortality was not reported due to data unavailability. Second, we are unable to show gender-specific mortality among COVID-19 patients with or without mortality. Third, we are also unable to show what would be the optimal dose for a protective effect against COVID-19. Fourth, our analyses were only based on retrospective observational studies. The quality of retrospective observational studies is generally poor and contains some risk of bias. However, there were no randomized controlled trials (RCTs) available while conducting this study. In future, RCTs should be conducted to confirm or refute their association. Fifth, there was also significant heterogeneity of the pooled studies, although we did use random effect models to reduce the bias of studies due to heterogeneity. Finally, our study could not provide any information about the risk of mortality of COVID-19 patients with continuation or discontinuation of metformin use until admission due to lack of data.

## 5. Conclusions

Our updated meta-analysis shows that metformin use is associated with a reduced risk of mortality among patients with COVID-19. However, the possibility of confounding factors cannot be excluded. Clinicians also need to carefully evaluate the actual benefits of metformin for patients who are currently taking it and who are also at risk of COVID-19 mortality. A large prospective RCT is warranted to assess the beneficial effects of metformin treatment in COVID-19, especially in nondiabetic patients.

## Figures and Tables

**Table 1:** Characteristics of included studies.

Author	Country	Design	Data Collection (2020)	Participants	COVID-19 Patients	No. of Metformin Users	Age	Male (%)	Patient Criteria	HR/OR	Outcome (within Days)
Abu-Jamous [16]	UK	Retrospective	1 January–27 May	5294	1253	21	N/R	N/R	Type-2	0.19 (0.05–0.70)	In-hospital (21 days)
Khunti [30]	UK	Retrospective	16 February–31 August	2,851,465	13,479 ^#^	1,800,005	Range	55.9	Type-2	0.77 (0.73–0.81)	In-hospital
Jiang [17]	China	Retrospective	31 December–31 March	328	328	100	65	44.6	Type-2	0.48 (0.13–1.74)	In-hospital (30 days)
Cheng [7]	China	Retrospective	1 January–17 March	1213	1213	687	N/R	N/R	Type-2	0.87 (0.36–2.12)	In-hospital (28 days)
Crouse [18]	USA	Retrospective	15 February–22 June	25,326	604	239	Range	45	Type-2	0.33 (0.13–0.84)	In-hospital
Ghany [19]	USA	Retrospective	1 January–14 August	1139	1139	392	70.9	39	Type-2	0.34 (0.19–0.59)	In-hospital
Gao [8]	China	Retrospective	31 January–20 March	2399	2399	56	Rang	39.3	Type-2	3.96(1.03–15.19)	In-hospital
Bramante [20]	USA	Retrospective	4 March–4 December	9555	9555	342	60.4	56.2	Type-2	0.32 (0.15–0.66)	In-hospital
Lalau [21]	France	Retrospective	10 March–10 April	2449	2449	1496	70.9	64	Type-2	0.46 (0.36–0.60)	In-hospital (28 days)
Lally [22]	USA	Retrospective	1 March–13 May	775	775	39	75.6	97.3	Type-2	0.48 (0.28, 0.84)	Nursing home (30 days)
Luo [23]	China	Retrospective	27 January–24 March	283	283	104	63	51	Type-2	0.23 (0.06–0.82)	In-hospital
Bramante [24]	USA	Retrospective	1 January–7 June	6256	6256	2333	73	51.6	Type-2	0·88 (0·78–1·00)	In-hospital
Oh [25]	Korea	Retrospective	1 January–4 June	122,040	8070	5946	Range	44.7	Type-2	1.26 (0.81, 1.95)	In-hospital
P. Belmonte [26]	Spain	Retrospective	1 March–19 July	2666	2666	465	74.9	61.9	Type-2	1.10 (0.76–1.60)	In-hospital
Wang ^#^[27]	UK	Retrospective	30 January–13 October	71,634	39,829	59,724	64.8	61.9	Type-2	0.74 (0.42, 1.32)	In-hospital
Wang [28]	USA	Retrospective	1 March–30 April	58	58	9	67	52	Type-2	0.35 (0.01–3.08)	In-hospital

^#^ = COVID-19 related deaths only, N/R = Not reported.

**Table 2:** Summary of subgroup analyses.

Subgroup	No. of Study	Effect Size	95% CI	*p*-Value	*I* ^2^	Q-Value	τ^2^
Nationwide/EHR	8	0.74	0.60–0.89	0.002	80.88	36.62	0.04
Hospital	8	0.45	0.27–0.74	0.002	58.48	16.86	0.26
North America	6	0.43	0.26–0.72	0.01	85.57	34.65	0.31
Europe	5	0.67	0.47–0.94	0.02	82.69	23.11	0.10
Asia	5	0.90	0.43–1.86	0.78	64.12	11.15	0.40

**Table 3:** Comparison of effect size with other studies.

Author	Number of Article Included	OR/RR with 95% CI	*p*-Value	Q	I^2^ (%)	τ^2^	*p*-Value	Subgroup Analysis
Scheen 2020 [34]	4	0.75 (0.67–0.85)	–	–	61	–	0.05	No
Hariyanto 2020 [32]	5	0.54 (0.32–0.90)	0.02	–	54	0.17	0.07	No
Kow 2020 [33]	5	0.62 (0.43–0.89)	–	5.62	29	–	0.23	No
Lukito 2020 [35]	9	0.64 (0.43–0.97)	0.03	52.1	–	–	–	No
Our study	16	0.65 (0.54–0.80)	<0.001	75.88	62.20	0.06	<0.001	Yes

**Table 4:** The beneficial mechanism of metformin against COVID-19.

Drug	Mechanism	References
Metformin	Improve glucose control	[46,47]
	Increase insulin sensitivity	[48,49]
	Improves low-grade inflammation in obesity	[50,51]
	Reduction in body weight	[52,53]
	Decrease inflammatory cytokines	[54,55]
	Decrease reactive oxygen species production	[56,57,58]
	Protective arm of the renin-angiotensin-aldosterone system (RAAS)	[49,59,60]
	Decrease oxidative stress	[61]
	Decrease fibrosis	[62]
	Decrease renal hypoxia	[63,64]
	Reduction in neutrophils	[65]
	Increased urinary sodium excretion and decrease NCC activity	[66]
	Increase autophagy and Sirt1/FoxO1 and decrease GBM thickness	[67]
	Reduce inflammatory marker release	[39]

NCC: Sodium-Chloride Cotransporter; FoxO1: Forkhead Box O1; GBM: Glioblastoma.

## Data Availability

Not applicable.

## Supplementary Materials

The following are available online at https://www.mdpi.com/article/10.3390/jcm10163507/s1, Figure S1: Searching strategy, Figure S2: Metformin use and the risk of mortality of patients with COVID-19 (Studies included metformin users less than 1000 participants), Figure S3: Metformin use and the risk of mortality of patients with COVID-19 (Studies included metformin users between 1000 and 10,000 participants), Figure S4: Metformin use and the risk of mortality of patients with COVID-19 (Studies included metformin users more than 10,000 participants).

## References

[1] Wang C., Horby P.W., Hayden F.G., Gao G.F. (2020). A novel coronavirus outbreak of global health concern. Lancet.

[2] Yao X., Ye F., Zhang M., Cui C., Huang B., Niu P., Liu X., Zhao L., Dong E., Song C. (2020). In vitro antiviral activity and projection of optimized dosing design of hydroxychloroquine for the treatment of severe acute respiratory syndrome coronavirus 2 (SARS-CoV-2). Clin. Infect. Dis..

[3] Poly T.N., Islam M.M., Yang H.C., Lin M.C., Jian W.-S., Hsu M.-H., Li Y.-C.J. (2021). Obesity and Mortality Among Patients Diagnosed With COVID-19: A Systematic Review and Meta-Analysis. Front. Med..

[4] Lippi G., Wong J., Henry B.M. (2020). Hypertension and its severity or mortality in Coronavirus Disease 2019 (COVID-19): A pooled analysis. Pol. Arch. Intern. Med..

[5] Kumar A., Arora A., Sharma P., Anikhindi S.A., Bansal N., Singla V., Khare S., Srivastava A. (2020). Is diabetes mellitus associated with mortality and severity of COVID-19? A meta-analysis. Diabetes Metab. Syndr. Clin. Res. Rev..

[6] Pranata R., Huang I., Lim M.A., Wahjoepramono E.J., July J. (2020). Impact of cerebrovascular and cardiovascular diseases on mortality and severity of COVID-19–systematic review, meta-analysis, and meta-regression. J. Stroke Cerebrovasc. Dis..

[7] Cheng X., Liu Y.-M., Li H., Zhang X., Lei F., Qin J.-J., Chen Z., Deng K.-Q., Lin L., Chen M.-M. (2020). Metformin Is Associated with Higher Incidence of Acidosis, but Not Mortality, in Individuals with COVID-19 and Pre-existing Type 2 Diabetes. Cell Metab..

[8] Gao Y., Liu T., Zhong W., Liu R., Zhou H., Huang W., Zhang W. (2020). Risk of Metformin in Patients with Type 2 Diabetes with COVID-19: A Preliminary Retrospective Report. Clin. Transl. Sci..

[9] Foretz M., Guigas B., Viollet B. (2019). Understanding the glucoregulatory mechanisms of metformin in type 2 diabetes mellitus. Nat. Rev. Endocrinol..

[10] Šestan M., Marinović S., Kavazovic I., Cekinović Đ., Wueest S., Wensveen T.T., Brizić I., Jonjic S., Konrad D., Wensveen F. (2018). Virus-Induced Interferon-γ Causes Insulin Resistance in Skeletal Muscle and Derails Glycemic Control in Obesity. Immunity.

[11] Andrzejewski S., Siegel P.M., St-Pierre J. (2018). Metabolic profiles associated with metformin efficacy in cancer. Front. Endocrinol..

[12] Siska P.J., Rathmell J.C. (2015). T cell metabolic fitness in antitumor immunity. Trends Immunol..

[13] Moher D., Liberati A., Tetzlaff J., Altman D.G. (2009). The PRISMA Group. Preferred Reporting Items for Systematic Reviews and Meta-Analyses: The PRISMA Statement. PLoS Med..

[14] Islam M., Iqbal U., Walther B., Atique S., Dubey N.K., Nguyen P.-A., Poly T.N., Masud J.H.B., Li Y.-C., Shabbir S.-A. (2016). Benzodiazepine Use and Risk of Dementia in the Elderly Population: A Systematic Review and Meta-Analysis. Neuroepidemiology.

[15] Wu C.C., Lee A.J., Su C.H., Huang C.Y., Islam M., Weng Y.C. (2021). Statin Use Is Associated with a Decreased Risk of Mortality among Patients with COVID-19. J. Clin. Med..

[16] Abu-Jamous B., Anisimovich A., Baxter J., Mackillop L., Vizcaychipi M.P., McCarthy A., Khan R.T. (2020). Associations of comorbidities and medications with COVID-19 outcome: A retrospective analysis of real-world evidence data. medRxiv.

[17] Jiang N., Chen Z., Liu L., Yin X., Yang H., Tan X., Wang J., Li H., Tian M., Lu Z. (2021). Association of metformin with mortality or ARDS in patients with COVID-19 and type 2 diabetes: A retrospective cohort study. Diabetes Res. Clin. Pr..

[18] Crouse A.B., Grimes T., Li P., Might M., Ovalle F., Shalev A. (2021). Metformin Use Is Associated with Reduced Mortality in a Diverse Population with COVID-19 and Diabetes. Front. Endocrinol..

[19] Ghany R., Palacio A., Dawkins E., Chen G., McCarter D., Forbes E., Chung B., Tamariz L. (2021). Metformin is associated with lower hospitalizations, mortality and severe coronavirus infection among elderly medicare minority patients in 8 states in USA. Diabetes Metab. Syndr. Clin. Res. Rev..

[20] Bramante C.T., Buse J., Tamaritz L., Palacio A., Cohen K., Vojta D., Liebovitz D., Mitchell N., Nicklas J., Lingvay I. (2021). Outpatient metformin use is associated with reduced severity of COVID-19 disease in adults with overweight or obesity. J. Med. Virol..

[21] Lalau J.-D., Al-Salameh A., Hadjadj S., Goronflot T., Wiernsperger N., Pichelin M., Allix I., Amadou C., Bourron O., Duriez T. (2021). Metformin use is associated with a reduced risk of mortality in patients with diabetes hospitalised for COVID-19. Diabetes Metab..

[22] Lally M.A., Tsoukas P., Halladay C.W., O’Neill E., Gravenstein S., Rudolph J.L. (2021). Metformin is associated with decreased 30-day mortality among nursing home residents infected with SARS-CoV2. J. Am. Med Dir. Assoc..

[23] Luo P., Qiu L., Liu Y., Liu X.-l., Zheng J.-l., Xue H.-y., Liu W.-h., Liu D., Li J. (2020). Metformin treatment was associated with decreased mortality in COVID-19 patients with diabetes in a retrospective analysis. Am. J. Trop. Med. Hyg..

[24] Bramante C.T., Ingraham N.E., Murray T.A., Marmor S., Hovertsen S., Gronski J., McNeil C., Feng R., Guzman G., Abdelwahab N. (2021). Metformin and risk of mortality in patients hospitalised with COVID-19: A retrospective cohort analysis. Lancet Health Longev..

[25] Oh T.K., Song I.-A. (2021). Metformin use and risk of COVID-19 among patients with type II diabetes mellitus: An NHIS-COVID-19 database cohort study. Acta Diabetol..

[26] Pérez-Belmonte L.M., Torres-Peña J.D., López-Carmona M.D., Ayala-Gutiérrez M.M., Fuentes-Jiménez F., Huerta L.J., Muñoz J.A., Rubio-Rivas M., Madrazo M., Garcia M.G. (2020). Mortality and other adverse outcomes in patients with type 2 diabetes mellitus admitted for COVID-19 in association with glucose-lowering drugs: A nationwide cohort study. BMC Med..

[27] Wang J., Cooper J.M., Gokhale K., Acosta-Mena D., Dhalla S., Byne N., Chandan J.S., Anand A., Okoth K., Subramanian A. (2021). Association of Metformin with Susceptibility to COVID-19 in People with Type 2 Diabetes. J. Clin. Endocrinol. Metab..

[28] Wang B., Van Oekelen O., Mouhieddine T.H., Del Valle D.M., Richter J., Cho H.J., Richard S., Chari A., Gnjatic S., Merad M. (2020). A tertiary center experience of multiple myeloma patients with COVID-19: Lessons learned and the path forward. J. Hematol. Oncol..

[29] Khunti K., Knighton P., Zaccardi F., Bakhai C., Barron E., Holman N., Kar P., Meace C., Sattar N., Sharp S. (2021). Prescription of glucose-lowering therapies and risk of COVID-19 mortality in people with type 2 diabetes: A nationwide observational study in England. Lancet Diabetes Endocrinol..

[30] Cai X.-L., Ji L.-N. (2019). Treatment response between Asian and non-Asian patients with type 2 diabetes: Is there any similarity or difference?. Chin. Med. J..

[31] Di Xiao J.Y., Zhang S.M., Liu R.R., Yin J.Y., Han X.Y., Li X., Zhang W., Chen X.P., Zhou H.H., Ji L.N. (2021). A Two-Stage Study Identifies Two Novel Polymorphisms in PRKAG2 Affecting Metformin Response in Chinese Type 2 Diabetes Patients. Pharm. Pers. Med..

[32] Hariyanto T.I., Kurniawan A. (2020). Metformin use is associated with reduced mortality rate from coronavirus disease 2019 (COVID-19) infection. Obes. Med..

[33] Kow C.S., Hasan S. (2021). Mortality risk with preadmission metformin use in patients with COVID-19 and diabetes: A meta-analysis. J. Med. Virol..

[34] Scheen A.J. (2020). Metformin and COVID-19: From cellular mechanisms to reduced mortality. Diabetes Metab..

[35] Lukito A.A., Pranata R., Henrina J., Lim M.A., Lawrensia S., Suastika K. (2020). The Effect of Metformin Consumption on Mortality in Hospitalized COVID-19 patients: A systematic review and meta-analysis. Diabetes Metab. Syndr. Clin. Res. Rev..

[36] Zangiabadian M., Nejadghaderi S.A., Zahmatkesh M.M., Hajikhani B., Mirsaeidi M., Nasiri M.J. (2021). The Efficacy and Potential Mechanisms of Metformin in the Treatment of COVID-19 in the Diabetics: A Systematic Review. Front. Endocrinol..

[37] Sukumar M., Liu J., Ji Y., Subramanian M., Crompton J.G., Yu Z., Roychoudhuri R., Palmer D.C., Muranski P., Karoly E.D. (2013). Inhibiting glycolytic metabolism enhances CD8+ T cell memory and antitumor function. J. Clin. Investig..

[38] Jung T.W., Lee M.W., Lee Y.J., Kim S.M. (2012). Metformin prevents endoplasmic reticulum stress-induced apoptosis through AMPK-PI3K-c-Jun NH2 pathway. Biochem. Biophys. Res. Commun..

[39] Isoda K., Young J.L., Zirlik A., MacFarlane L.A., Tsuboi N., Gerdes N., Schonbeck U., Libby P. (2006). Metformin inhibits proinflammatory responses and nuclear factor-κB in human vascular wall cells. Arterioscler. Thromb. Vasc. Biol..

[40] Siri M., Dastghaib S., Zamani M., Rahmani-Kukia N., Geraylow K.R., Fakher S., Keshvarzi F., Mehrbod P., Ahmadi M., Mokarram P. (2021). Autophagy, Unfolded Protein Response, and Neuropilin-1 Cross-Talk in SARS-CoV-2 Infection: What Can Be Learned from Other Coronaviruses. Int. J. Mol. Sci..

[41] Kaneto H., Kimura T., Obata A., Shimoda M., Kaku K. (2021). Multifaceted Mechanisms of Action of Metformin Which Have Been Unraveled One after Another in the Long History. Int. J. Mol. Sci..

[42] Groen B.B.L., Hamer H.M., Snijders T., Van Kranenburg J., Frijns D., Vink H., van Loon L.J. (2014). Skeletal muscle capillary density and microvascular function are compromised with aging and type 2 diabetes. J. Appl. Physiol..

[43] Yamaoka-Tojo M. (2020). Endothelial glycocalyx damage as a systemic inflammatory microvascular endotheliopathy in COVID-19. Biomed. J..

[44] Eskens B.J.M., Zuurbier C.J., Van Haare J., Vink H., Van Teeffelen J.W.G.E. (2013). Effects of two weeks of metformin treatment on whole-body glycocalyx barrier properties in db/db mice. Cardiovasc. Diabetol..

[45] Targosz-Korecka M., Malek-Zietek K.E., Kloska D., Rajfur Z., Stepien E.Ł., Grochot-Przeczek A., Szymonski M. (2020). Metformin attenuates adhesion between cancer and endothelial cells in chronic hyperglycemia by recovery of the endothelial glycocalyx barrier. Biochim. Biophys. Acta (BBA) Gen. Subj..

[46] Ceriello A. (2020). Hyperglycemia and the worse prognosis of COVID-19. Why a fast blood glucose control should be mandatory. Diabetes Res. Clin. Pract..

[47] Ceriello A., De Nigris V., Prattichizzo F. (2020). Why is hyperglycaemia worsening COVID-19 and its prognosis?. Diabetes Obes. Metab..

[48] Chen Y., Gu F., Guan J.-L. (2018). Metformin Might Inhibit Virus through Increasing Insulin Sensitivity. Chin. Med. J..

[49] Sharma S., Ray A., Sadasivam B. (2020). Metformin in COVID-19: A possible role beyond diabetes. Diabetes Res. Clin. Pr..

[50] Jing Y., Wu F., Li D., Yang L., Li Q., Li R. (2018). Metformin improves obesity-associated inflammation by altering macrophages polarization. Mol. Cell. Endocrinol..

[51] Desai N., Roman A., Rochelson B., Gupta M., Xue X., Chatterjee P.K., Tam H.T., Metz C.N. (2013). Maternal metformin treatment decreases fetal inflammation in a rat model of obesity and metabolic syndrome. Am. J. Obstet. Gynecol..

[52] El-Arabey A.A., Abdalla M. (2020). Metformin and COVID-19: A novel deal of an old drug. J. Med. Virol..

[53] Seifarth C., Schehler B., Schneider H.J. (2012). Effectiveness of Metformin on Weight Loss in Non-Diabetic Individuals with Obesity. Exp. Clin. Endocrinol. Diabetes.

[54] Ansari G., Mojtahedzadeh M., Kajbaf F., Najafi A., Khajavi M.R., Khalili H., Rouini M.R., Ahmadi H., Abdollahi M. (2008). How does blood glucose control with metformin influence intensive insulin protocols? Evidence for involvement of oxidative stress and inflammatory cytokines. Adv. Ther..

[55] Chen W., Liu X., Ye S. (2016). Effects of metformin on blood and urine pro-inflammatory mediators in patients with type 2 diabetes. J. Inflamm..

[56] Algire C., Moiseeva O., Deschênes-Simard X., Amrein L., Petruccelli L., Birman E., Viollet B., Ferbeyre G., Pollak M.N. (2012). Metformin Reduces Endogenous Reactive Oxygen Species and Associated DNA Damage. Cancer Prev. Res..

[57] Ouslimani N., Peynet J., Bonnefont-Rousselot D., Thérond P., Legrand A., Beaudeux J.-L. (2005). Metformin decreases intracellular production of reactive oxygen species in aortic endothelial cells. Metabolism.

[58] Hou X., Song J., Li X.-N., Zhang L., Wang X., Chen L., Shen Y.H. (2010). Metformin reduces intracellular reactive oxygen species levels by upregulating expression of the antioxidant thioredoxin via the AMPK-FOXO3 pathway. Biochem. Biophys. Res. Commun..

[59] Malhotra A., Hepokoski M., McCowen K.C., Shyy J.Y.-J. (2020). ACE2, Metformin, and COVID-19. iScience.

[60] Nesti L., Natali A. (2017). Metformin effects on the heart and the cardiovascular system: A review of experimental and clinical data. Nutr. Metab. Cardiovasc. Dis..

[61] Borges C.M., Fujihara C.K., Malheiros D.M.A.C., De Ávila V.F., Formigari G.P., De Faria J.B.L. (2020). Metformin arrests the progression of established kidney disease in the subtotal nephrectomy model of chronic kidney disease. Am. J. Physiol. Physiol..

[62] Sturmlechner I., Durik M., Sieben C.J., Baker D.J., Van Deursen J.M. (2016). Cellular senescence in renal ageing and disease. Nat. Rev. Nephrol..

[63] Christensen M., Schiffer T., Gustafsson H., Krag S.P., Nørregaard R., Palm F. (2019). Metformin attenuates renal medullary hypoxia in diabetic nephropathy through inhibition uncoupling protein-2. Diabetes/Metab. Res. Rev..

[64] Jiang X., Ruan X.-l., Xue Y.-x., Yang S., Shi M., Wang L.-n. (2020). Metformin reduces the senescence of renal tubular epithelial cells in diabetic nephropathy via the MBNL1/miR-130a-3p/STAT3 pathway. Oxidative Med. Cell. Longev..

[65] Dalan R. (2020). Metformin, neutrophils and COVID-19 infection. Diabetes Res. Clin. Pract..

[66] Hashimoto H., Nomura N., Shoda W., Isobe K., Kikuchi H., Yamamoto K., Fujimaru T., Ando F., Mori T., Okado T. (2018). Metformin increases urinary sodium excretion by reducing phosphorylation of the sodium-chloride cotransporter. Metabolism.

[67] Xu J., Liu L., Xu L., Xing Y., Ye S. (2020). Metformin alleviates renal injury in diabetic rats by inducing Sirt1/FoxO1 autophagic signal axis. Clin. Exp. Pharmacol. Physiol..

