# Supplementary material for: Metformin Use Is Associated with Decreased Mortality in COVID-19 Patients with Diabetes: Evidence from Retrospective Studies and Biological Mechanism
Source: J Clin Med. 2021 Aug 9;10(16):3507. doi: 10.3390/jcm10163507 (PMC8397144; doi:10.3390/jcm10163507)
Supplement: Supplementary file 1 [file jcm-10-03507-s001.zip › jcm-1292289-supplementary.pdf]

PRISMA 2009 Flow Diagram

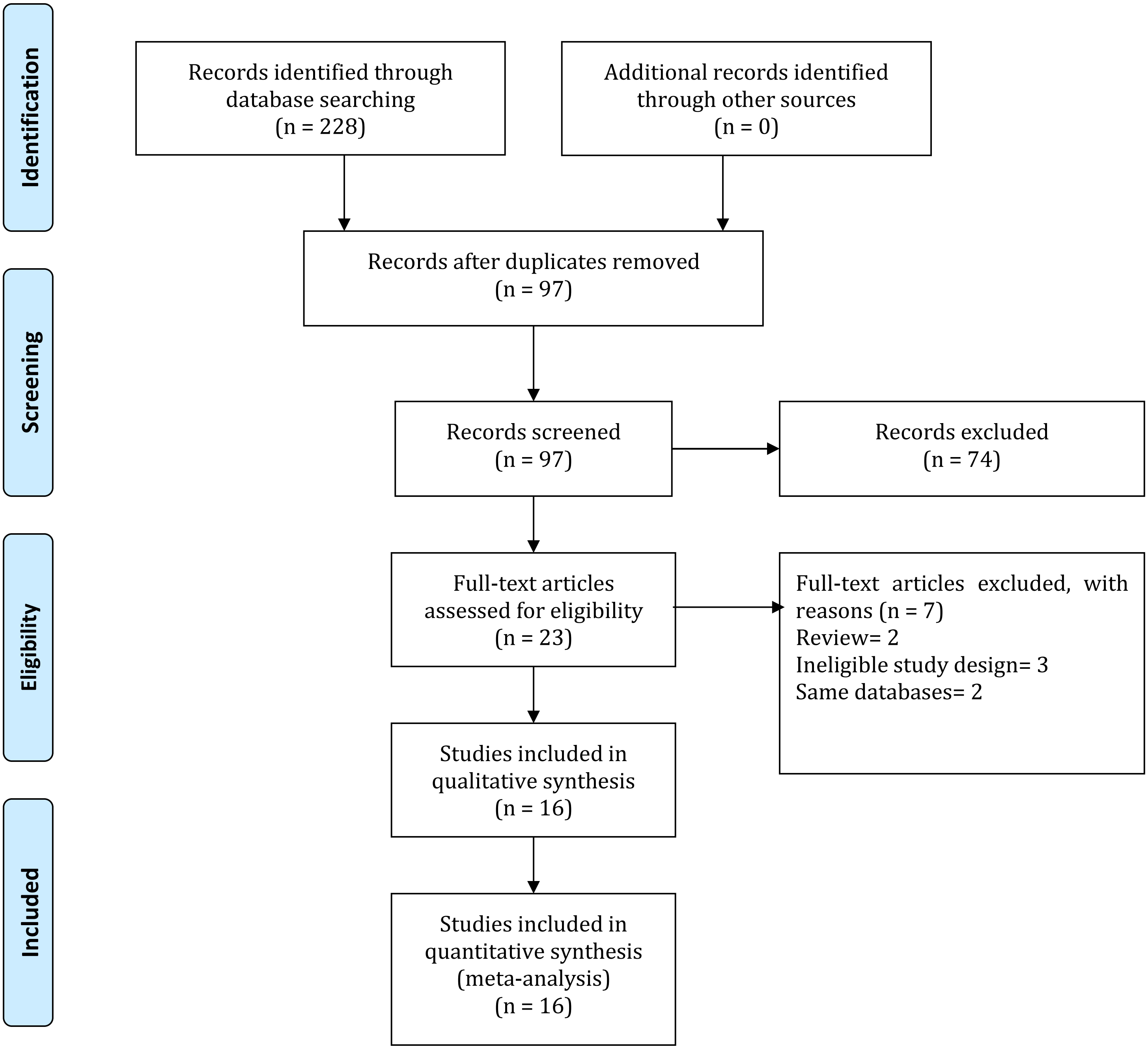

Supplementary Figure S1. Searching strategy.

Study name	Risk ratio	Lower limit	Upper limit	Z-Value	p-Value	Relative weight
Abu-Jamous-2020	0.190	0.051	0.711	-2.467	0.014	6.79
Jiang-2021	0.480	0.131	1.756	-1.109	0.267	6.92
Cheng-2020	0.870	0.359	2.111	-0.308	0.758	9.79
Crouse-2021	0.330	0.130	0.839	-2.329	0.020	9.42
Ghany-2021	0.340	0.193	0.599	-3.732	0.000	12.48
Gao-2020	3.960	1.031	15.207	2.005	0.045	6.64
Bramante-2021	0.320	0.153	0.671	-3.015	0.003	11.00
Lally-2021	0.480	0.277	0.831	-2.619	0.009	12.63
Luo-2020	0.230	0.062	0.850	-2.203	0.028	6.86
Belmonte-2021	1.100	0.758	1.596	0.502	0.616	13.99
Wang-2020	0.350	0.040	3.071	-0.947	0.343	3.47
**Random effect:**	0.513	0.324	0.813	-2.843	0.004	

Heterogeneity (I^2): 69.31, Q=32.58, τ^2=0.35, p<0.001

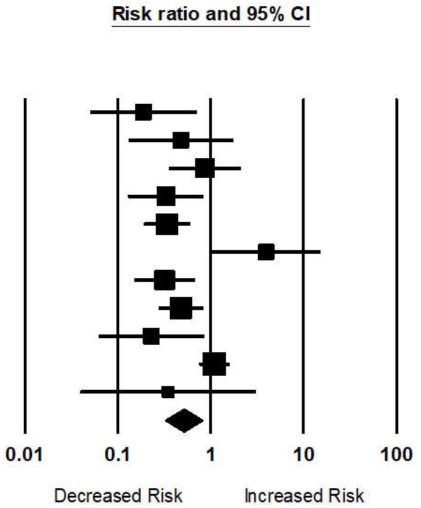

Figure S2. Metformin use and the risk of mortality of patients with COVID-19 (Studies included metformin users less than 1000 participants).

Study name	Risk ratio	Lower limit	Upper limit	Z-Value	p-Value	Relative weight
Lalau-2021	0.460	0.356	0.594	-5.959	0.000	34.19
Bramante*-2021	0.880	0.777	0.996	-2.017	0.044	36.64
Oh-2021	1.260	0.812	1.955	1.031	0.302	29.17
**Random effect:**	0.783	0.473	1.296	-0.952	0.341	

Heterogeneity (I^2): 91.80, Q=24.40, τ^2=0.17, p<0.001

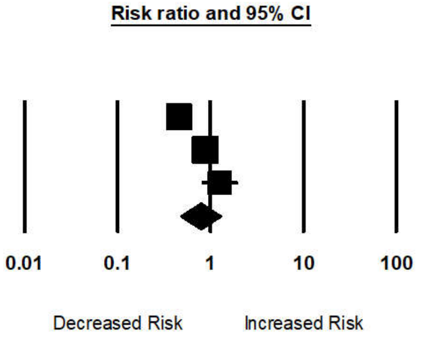

Figure S3. Metformin use and the risk of mortality of patients with COVID-19 (Studies included metformin users between 1000 and 10,000 participants).

Study name	Risk ratio	Lower limit	Upper limit	Z-Value	p-Value	Relative weight
Khunti-2021	0.770	0.731	0.811	-9.852	0.000	99.18
Wang-2021	0.740	0.417	1.312	-1.031	0.303	0.82
**Random effect**	0.770	0.731	0.811	-9.905	0.000	

Heterogeneity (I^2): 0, Q=0.01, τ^2=0, p=0.89

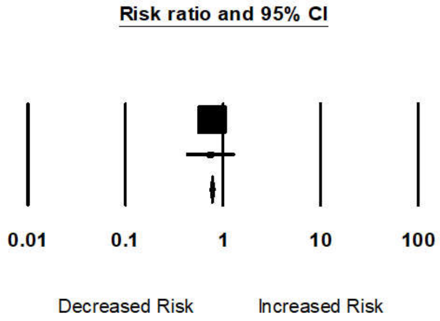

Figure S4. Metformin use and the risk of mortality of patients with COVID-19 (Studies included metformin users more than 10,000 participants).
